# Influence of spectral shaping and tube voltage modulation in ultralow-dose computed tomography of the abdomen

**DOI:** 10.1186/s12880-024-01228-1

**Published:** 2024-02-23

**Authors:** Philipp Feldle, Jan-Peter Grunz, Andreas Steven Kunz, Pauline Pannenbecker, Theresa Sophie Patzer, Svenja Pichlmeier, Stephanie Tina Sauer, Robin Hendel, Süleyman Ergün, Thorsten Alexander Bley, Henner Huflage

**Affiliations:** 1 Department of Diagnostic and Interventional Radiology, University Hospital Würzburg, Oberdürrbacher Straße 6, 97080 Würzburg, Germany; 2 Institute of Anatomy and Cell Biology, University of Würzburg, Koellikerstraße 6, 97070 Würzburg, Germany

**Keywords:** Spectral shaping, Tin prefiltration, Abdominal imaging, Dose reduction

## Abstract

**Purpose:**

Unenhanced abdominal CT constitutes the diagnostic standard of care in suspected urolithiasis. Aiming to identify potential for radiation dose reduction in this frequent imaging task, this experimental study compares the effect of spectral shaping and tube voltage modulation on image quality.

**Methods:**

Using a third-generation dual-source CT, eight cadaveric specimens were scanned with varying tube voltage settings with and without tin filter application (Sn 150, Sn 100, 120, 100, and 80 kVp) at three dose levels (3 mGy: standard; 1 mGy: low; 0.5 mGy: ultralow). Image quality was assessed quantitatively by calculation of signal-to-noise ratios (SNR) for various tissues (spleen, kidney, trabecular bone, fat) and subjectively by three independent radiologists based on a seven-point rating scale (7 = excellent; 1 = very poor).

**Results:**

Irrespective of dose level, Sn 100 kVp resulted in the highest SNR of all tube voltage settings. In direct comparison to Sn 150 kVp, superior SNR was ascertained for spleen (*p* ≤ 0.004) and kidney tissue (*p* ≤ 0.009). In ultralow-dose scans, subjective image quality of Sn 100 kVp (median score 3; interquartile range 3–3) was higher compared with conventional imaging at 120 kVp (2; 2–2), 100 kVp (1; 1–2), and 80 kVp (1; 1–1) (all *p* < 0.001). Indicated by an intraclass correlation coefficient of 0.945 (95% confidence interval: 0.927–0.960), interrater reliability was excellent.

**Conclusions:**

In abdominal CT with maximised dose reduction, tin prefiltration at 100 kVp allows for superior image quality over Sn 150 kVp and conventional imaging without spectral shaping.

## Introduction

Prevalence of kidney stone disease has increased globally over the last decades, even among children [1–3]. Its high rate of reoccurrence is well documented, often requiring repeated imaging by computed tomography (CT), which is nowadays considered the diagnostic modality of choice [4, 5]. Although CT outperforms ultrasound with regards to sensitivity and specificity, focus has shifted towards concerns about radiation dose and possible long term side effects for years [6–9].

While the detection of urolithiasis constitutes a high-contrast imaging task, abdominal imaging in general is challenged by the low-contrast properties of parenchymal organs. For CT angiography, low tube voltage imaging is an established approach to increase contrast due to the photoelectric effect [10]. Notably, some studies suggest a similar advantage at low tube voltages in non-contrast examinations [11]. While the relationship between radiation dose and tube current is known to be linear, i.e., doubling the tube current results in doubling the dose, the relationship between dose and tube voltage is proportional to the square of the voltage. Thus, a tube voltage reduction from 120 to 100 kVp is associated with a 33% reduction in radiation dose, and a reduction to 80 kVp with a decrease by 65% [12]. However, a disadvantage of lower tube voltage lies in the reduced tissue penetration of photons and resulting increase of image noise and higher dependency on patient size [12, 13]. Whether lower tube currents allow for maintaining sufficient image quality particularly depends on the diagnostic task [12, 13].

In the last decade, the application of tin filters has attracted increasing attention as spectral shaping allows for considerable dose savings, particularly for imaging of the lungs und paranasal sinus [14–16]. Quantifying the dose reduction potential of spectral shaping remains difficult, however, as the results of the individual studies vary considerably [17–19]. Since the use of tin filters is also becoming increasingly common for abdominal CT examinations in recent years [18, 20, 21], a direct comparison between different dose saving techniques is warranted.

As excessive radiation reduction may render an examination diagnostically insufficient, this cadaveric study aimed to determine the most efficient ultralow-dose scan protocol for unenhanced abdominal CT. In order to achieve that, low-kV imaging was compared with tin filtration protocols at two different tube voltages on a third-generation dual-source CT system.

## Methods

For this experimental investigation, scans were performed on eight formalin-fixed cadaveric specimens obtained from the local university’s anatomical institute. As body donors had volunteered their corpses for scientific and educational purposes during their lifetime, no further written informed consent was required. Approval for the study was granted by the local ethics committee.

### Scan and image parameters

All examinations were performed on a commercially available third-generation dual-source CT scanner (Somatom Force, Siemens Healthcare GmbH, Forchheim, Germany) with an energy-integrating detector system. Specimens were examined in supine position with elevated arms. Scouts were acquired with identical dose settings in anterior-posterior orientation. Each specimen was scanned with five different tube settings, of which two employed spectral shaping with a 0.6 mm tin filter (Sn 100 kVp, Sn 150 kVp). The other settings used tube voltages of 120, 100 and 80 kVp. With each tube setting used for scans at three different dose levels, a total of 15 CT examinations were performed on each specimen. Target volume computed tomography dose indices (CTDI_vol_) were 3 mGy for standard-dose, 1 mGy for low-dose, and 0.5 mGy for ultralow-dose examinations. Automatic dose modulation was activated for all scans, as is mandatory in clinical routine. Without repositioning of specimens, scan ranges were set with identical length in all 15 consecutive scans. All datasets were reconstructed with a field of view of 350 mm, a slice thickness of 3 mm and a regular soft tissue body kernel (Br36) employing iterative reconstruction at a strength level of 3. (Admire, Siemens Healthcare GmbH). Window settings were selected at 400 (width) / 50 (centre) Hounsfield units, while readers were allowed to adjust according to personal preferences.

### Objective image quality

Standardized regions of interest were placed in the spleen, renal cortex, vertebral body and fat tissue at kidney level, each, on three consecutive slices. Mean density in Hounsfield units and standard deviation was measured to calculate a signal-to-noise ratio (SNR) for each organ according to the following formula:

$$ SNR=\frac{mean\, attenuation \left(organ\, tissue\right) }{standard\, deviation \left(organ\, tissue\right)}$$

### Subjective image quality

All datasets were independently analysed by three radiologists with at least five years of experience in CT imaging using a commercially available PACS system (Merlin, Phönix-PACS, Freiburg, Germany) and diagnostic monitors (30-inch diameter, RadiForce RX660, EIZO, Hakusan, Japan). For each dataset, readers assessed image quality according to a seven-point scale: 1 = very poor; 2 = poor; 3 = fair; 4 = satisfactory; 5 = good; 6 = very good; 7 = excellent.

### Statistics

The statistical analysis was conducted using specialized software (IBM SPSS Statistics, Armonk, USA). Kolmogorov-Smirnov tests were used to examine the normal distribution of cardinal variables. If variables were normally distributed, they are presented as mean ± standard deviation. Nonparametric variables are reported as absolute and relative frequencies, along with median values and interquartile ranges. Friedman tests were employed to compare ordinal-scaled variables, while one-way repeated measures ANOVA was used for comparing continuous data. Pairwise post-hoc tests were conducted with Bonferroni adjustment to account for multiple comparisons. The intraclass correlation coefficient (ICC) was calculated based on absolute agreement of single measures using a two-way random effects model. The ICC values were categorized according to established guidelines [22]. Statistical significance was determined at *p*-values of less than 0.05.

## Results

### Radiation dose

With automatic exposure control activated, the standard-dose protocols were associated with a mean CTDI_vol_ of 2.93 mGy ± 0.49 mGy across the performed 40 scans. While the low-dose protocols resulted in a mean CTDI_vol_ of 0.93 mGy ± 0.19 mGy, the ultralow-dose protocols resulted in a mean CTDI_vol_ of 0.47 ± 0.09 mGy. No substantial difference in radiation dose was ascertained for the five individual tube voltage settings on each dose level (all *p* > 0.05). Table 1 provides a detailed display of the radiation dose administered in each specimen.

**Table 1:** Radiation dose for each cadaveric specimen with different acquisition protocols employing automatic exposure control

Tube voltage	Sn 100 kVp			Sn 150 kVp			120 kVp			100 kVp			80 kVp		
Dose level	Standard	Low	Ultralow	Standard	Low	Ultralow	Standard	Low	Ultralow	Standard	Low	Ultralow	Standard	Low	Ultralow
Specimen 1	3.40	1.12	0.54	3.69	1.25	0.61	3.91	1.27	0.60	3.71	1.26	0.65	3.56	1.34	0.7
Specimen 2	3.00	0.99	0.47	3.28	1.06	0.5	3.51	1.10	0.47	3.39	1.08	0.49	2.79	0.94	0.47
Specimen 3	3.18	1.05	0.47	3.07	1.10	0.53	3.48	1.10	0.51	3.29	1.18	0.53	2.9	1.00	0.48
Specimen 4	2.61	0.80	0.39	2.66	0.76	0.37	2.91	0.94	0.40	2.87	0.90	0.36	2.62	0.77	0.54
Specimen 5	3.12	1.05	0.48	3.35	1.14	0.57	3.38	0.94	0.53	3.37	1.10	0.6	3.40	1.29	0.55
Specimen 6	2.75	0.95	0.41	2.87	0.98	0.48	2.81	0.77	0.47	2.79	0.98	0.48	2.72	1.08	0.53
Specimen 7	2.24	0.76	0.34	1.97	0.72	0.36	2.17	0.64	0.38	2.19	0.72	0.37	2.16	0.73	0.36
Specimen 8	2.40	0.82	0.35	2.37	0.79	0.39	2.34	0.67	0.38	2.39	0.82	0.39	2.44	0.83	0.41
**CTDI**_**vol**_ **[mGy]**	2.84 ± 0.38	0.94 ± 0.13	0.43 ± 0.07	2.91 ± 0.52	0.98 ± 0.18	0.48 ± 0.09	3.06 ± 0.57	0.93 ± 0.21	0.47 ± 0.07	3.00 ± 0.50	1.01 ± 0.17	0.48 ± 0.10	2.82 ± 0.44	1.00 ± 0.21	0.51 ± 0.10

***Note.– CTDI***_***vol***_ *– volume computed tomography dose index*

### Objective image quality

**Fig. 1:**
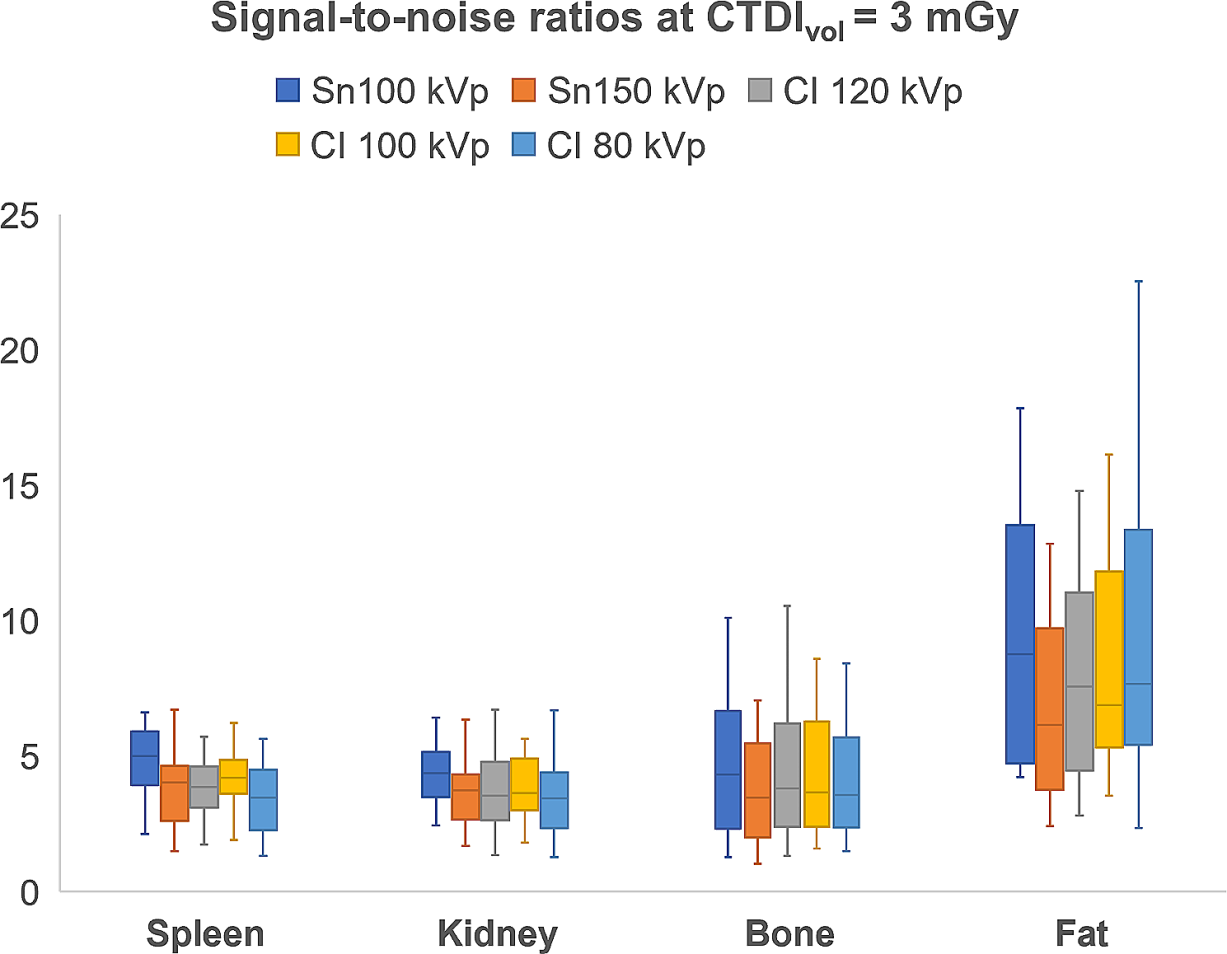
Boxplots illustrate differences in quantitative image quality assessment. At a standard dose level (CTDI_vol_ = 3 mGy), image acquisition with tin filtration at 100 kVp generated the highest signal-to-noise levels of all scan protocols in every tissue ***Note***. ***CI****– conventional imaging protocol without tin prefiltration*

**Fig. 2:**
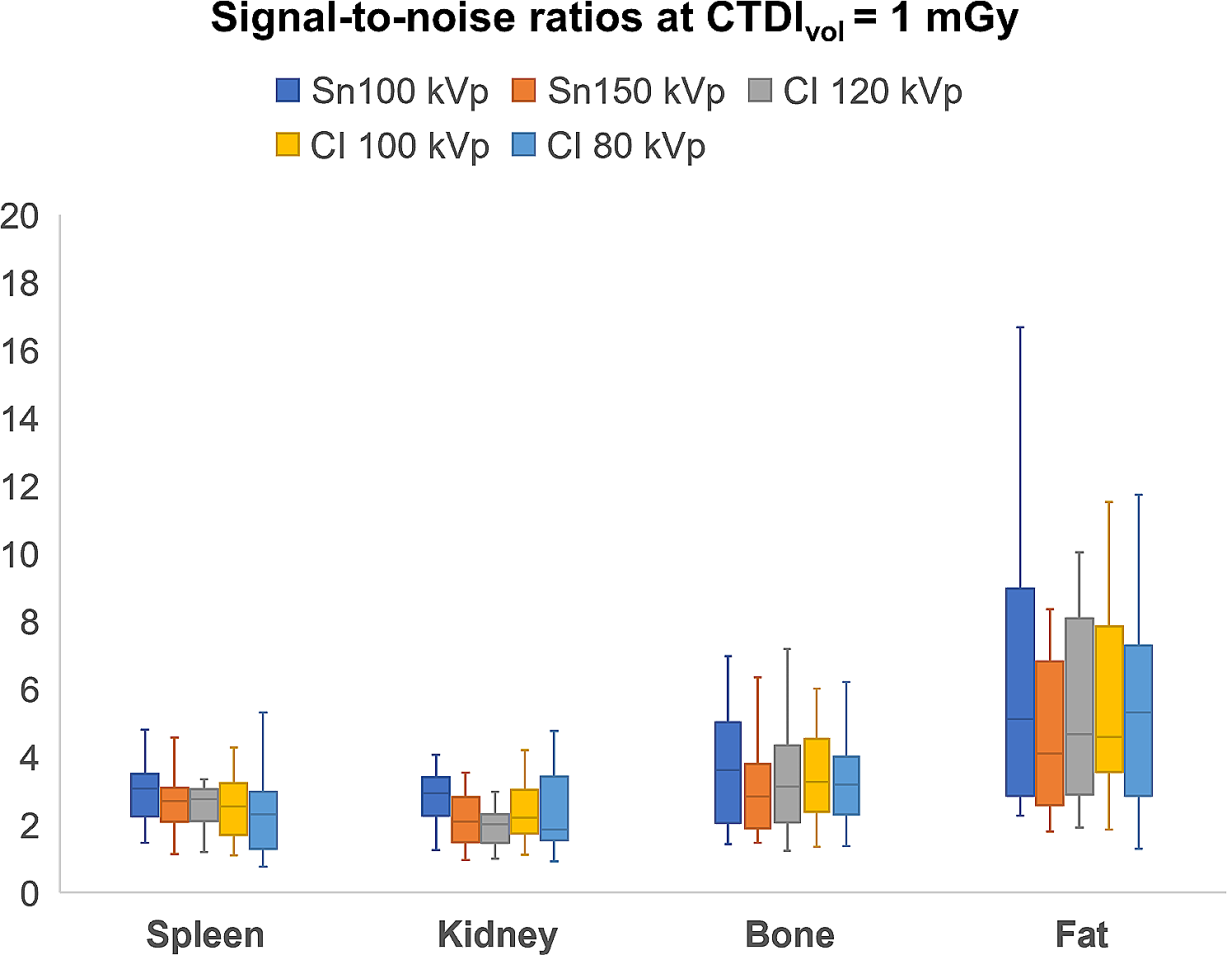
Irrespective of tissue, images acquired with Sn 100 kVp resulted in the highest signal-to-noise ratio at a low dose level (CTDI_vol_ = 1 mGy) ***Note***. ***CI****– conventional imaging protocol without tin prefiltration*

**Fig. 3:**
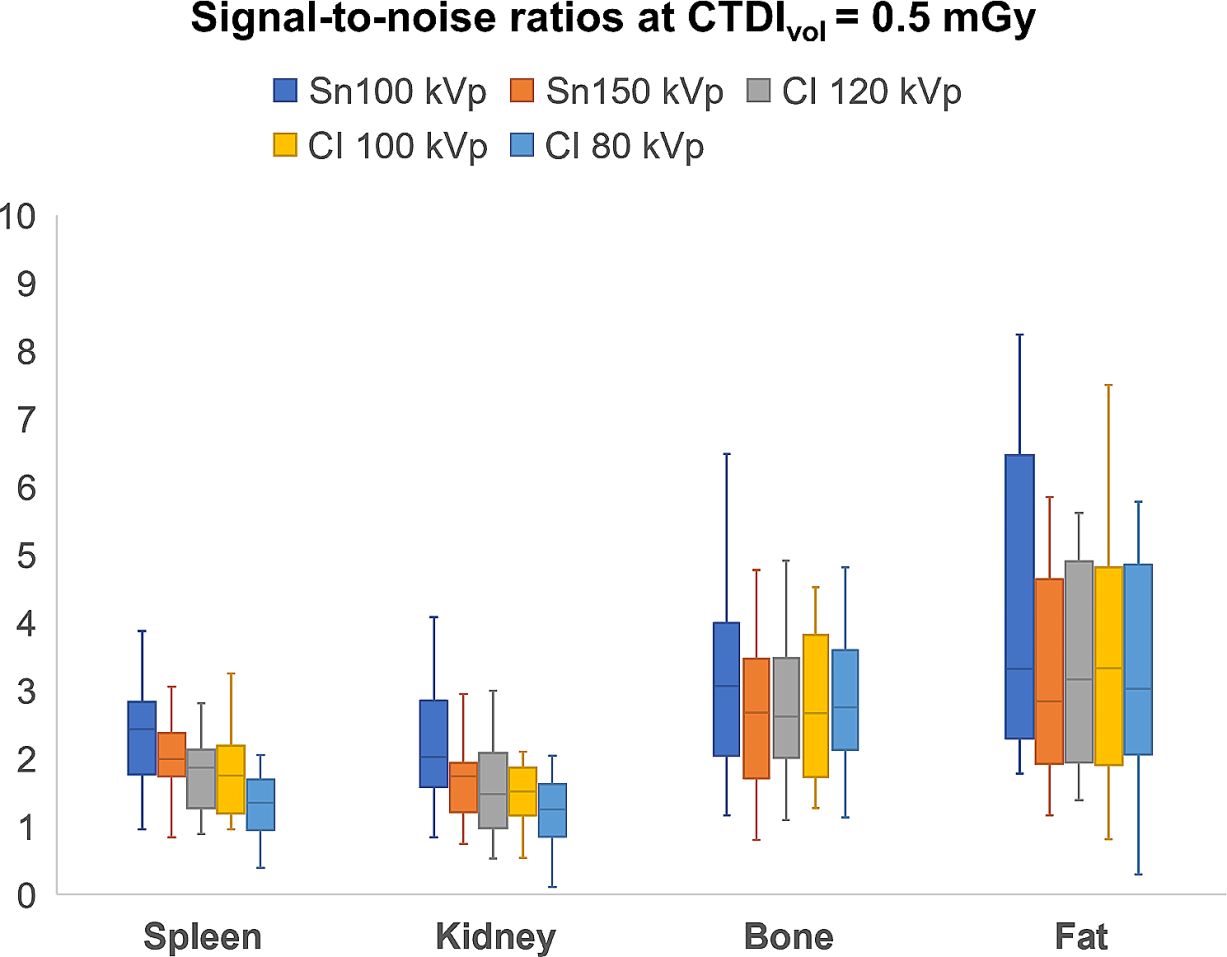
At an ultralow dose level (CTDI_vol_ = 0.5 mGy), spectral shaping at 100 kVp produced the highest SNR levels for each investigated tissue ***Note***. ***CI****– conventional imaging protocol without tin prefiltration*

While Sn 100 kVp provided the highest SNR within each dose level, SNR decreased with lower applied CTDI_vol_. In direct comparison to Sn 150 kVp, superior SNR was ascertained for Sn 100 kVp in spleen (*p* ≤ 0.004) and kidney tissue (*p* ≤ 0.009) at all three dose levels. In contrast, no significant difference could be ascertained among the tin prefiltration protocols for bone and fat tissue. Detailed SNR comparisons between all protocols in every tissue are provided in Fig. 1 for standard dose, in Fig. 2 for low-dose, and in Fig. 3 for ultralow-dose imaging. A comprehensive overview of SNR values is provided in Table 2.

**Table 2:** Signal-to-noise ratios were calculated for all scan protocols and tissues. Results are presented as mean values ± standard deviations

Tissue	Tube voltage	Standard-dose protocol	Low-dose protocol	Ultralow-dose protocol
**Spleen**	Sn 100 kVp	4.74 ± 1.32	3.04 ± 0.92	2.33 ± 0.77
	Sn 150 kVp	3.76 ± 1.22	2.54 ± 0.83	1.98 ± 0.55
	120 kVp	3.88 ± 1.26	2.68 ± 0.84	1.73 ± 0.52
	100 kVp	4.04 ± 1.14	2.54 ± 0.89	1.78 ± 0.60
	80 kVp	3.42 ± 1.25	2.25 ± 1.04	1.33 ± 0.47
**Kidney**	Sn 100 kVp	4.45 ± 1.22	2.79 ± 0.74	2.20 ± 0.82
	Sn 150 kVp	3.57 ± 1.13	2.17 ± 0.72	1.66 ± 0.53
	120 kVp	3.67 ± 1.35	1.96 ± 0.63	1.57 ± 0.64
	100 kVp	3.84 ± 1.43	2.37 ± 0.87	1.47 ± 0.41
	80 kVp	3.45 ± 1.30	2.36 ± 1.11	1.22 ± 0.46
**Bone**	Sn 100 kVp	4.61 ± 2.36	3.88 ± 1.97	3.26 ± 1.49
	Sn 150 kVp	3.97 ± 2.24	3.34 ± 1.95	2.85 ± 1.66
	120 kVp	4.28 ± 2.18	3.40 ± 1.58	3.03 ± 1.76
	100 kVp	4.24 ± 2.04	3.54 ± 1.57	2.91 ± 1.37
	80 kVp	4.05 ± 1.83	3.43 ± 1.64	2.98 ± 1.37
**Fat**	Sn 100 kVp	10.19 ± 6.19	6.44 ± 4.10	5.00 ± 4.41
	Sn 150 kVp	8.24 ± 7.70	4.62 ± 2.17	3.51 ± 2.35
	120 kVp	8.08 ± 3.74	5.90 ± 4.54	3.61 ± 1.97
	100 kVp	9.47 ± 5.91	6.12 ± 4.48	4.21 ± 4.08
	80 kVp	9.09 ± 4.96	5.67 ± 3.15	3.45 ± 1.94

### Subjective image quality

**Fig. 4:**
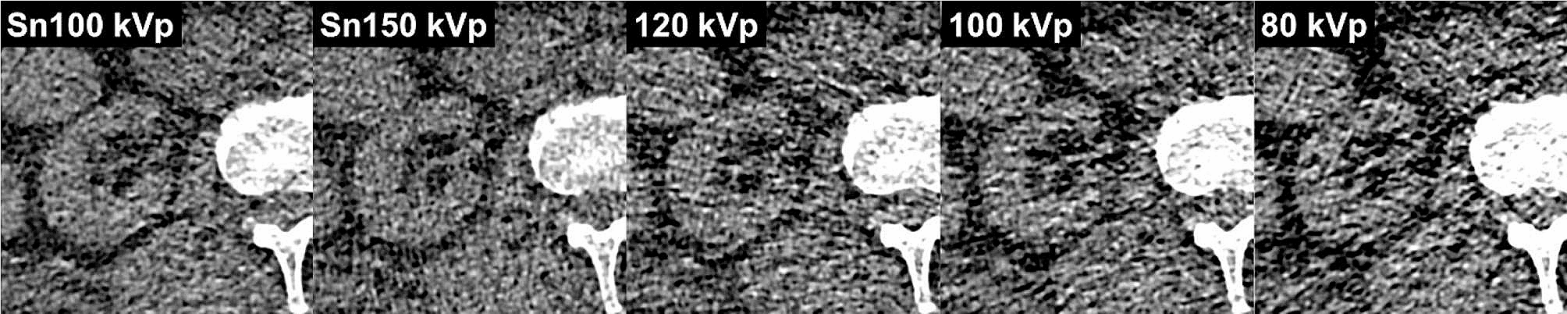
Ultralow-dose images of a cadaveric specimen’s right kidney (CTDI_vol_ = 0.5 mGy) with all investigated tube voltage settings. Note the increased image noise and pronounced hypodense streak artifacts due to photon starvation in conventional images without spectral shaping, particularly evident at 80 kVp (right image)

Higher radiation dose resulted in superior image quality ratings regardless of tube voltage setting. For each dose level, Friedmans rank-based ANOVA revealed significant differences between the five individual scan settings (all *p* < 0.008). On a standard dose level, pairwise post-hoc analyses demonstrated superior image quality of Sn 100 kVp (median score 7; interquartile range 6–7; *p* = 0.001) and Sn 150 kVp (6; 6–7; *p* = 0.019) compared to 80 kVp imaging (6; 6–6). Only Sn 100 kVp was found to be superior to 100 kVp (*p* = 0.009), however. In ultralow-dose scans, subjective image quality of Sn 100 kVp (3; 3–3) was deemed higher compared to conventional imaging at 120 kVp (2; 2–2), 100 kVp (1; 1–2), and 80 kVp (1; 1–1) (all *p* < 0.001). Sn 150 kVp (3; 2–3) was considered superior to 100 kVp and 80 kVp imaging (both *p* < 0.001), albeit equal to 120 kVp (*p* = 0.071). Among the three tube settings without spectral shaping, no significant difference was ascertained at any dose level (*p* ≥ 0.156). Detailed results of the subjective image quality analyses are given in Table 3. Figure 4 provides representative CT images for the five different tube settings at an ultralow-dose level of 0.5 mGy. An ICC of 0.945 (95% confidence interval: 0.927–0.960) indicated excellent interrater reliability.

**Table 3:** Pooled image quality assessment three radiologists. Results are displayed as absolute frequencies and percentages in parentheses. Median values are provided with interquartile ranges

Tube voltage	Sn 100 kVp			Sn 150 kVp			120 kVp			100 kVp			80 kVp		
Dose level	Standard	Low	Ultralow	Standard	Low	Ultralow	Standard	Low	Ultralow	Standard	Low	Ultralow	Standard	Low	Ultralow
7	13 (54.2)	0 (0)	0 (0)	9 (37.5)	0 (0)	0 (0)	6 (25.0)	0 (0)	0 (0)	0 (0)	0 (0)	0 (0)	0 (0)	0 (0)	0 (0)
6	11 (45.8)	0 (0)	0 (0)	15 (62.5)	0 (0)	0 (0)	18 (75.0)	0 (0)	0 (0)	21 (87.5)	0 (0)	0 (0)	18 (75.0)	0 (0)	0 (0)
5	0 (0)	20 (83.3)	0 (0)	0 (0)	18 (75.0)	0 (0)	0 (0)	17 (70.8)	0 (0)	3 (12.5)	14 (58.3)	0 (0)	6 (25.0)	9 (37.5)	0 (0)
4	0 (0)	4 (16.7)	5 (20.8)	0 (0)	6 (25.0)	0 (0)	0 (0)	7 (29.2)	0 (0)	0 (0)	10 (41.7)	0 (0)	0 (0)	15 (62.5)	0 (0)
3	0 (0)	0 (0)	19 (79.2)	0 (0)	0 (0)	15 (62.5)	0 (0)	0 (0)	0 (0)	0 (0)	0 (0)	0 (0)	0 (0)	0 (0)	0 (0)
2	0 (0)	0 (0)	0 (0)	0 (0)	0 (0)	9 (37.5)	0 (0)	0 (0)	20 (83.3)	0 (0)	0 (0)	7 (29.2)	0 (0)	0 (0)	6 (25.0)
1	0 (0)	0 (0)	0 (0)	0 (0)	0 (0)	0 (0)	0 (0)	0 (0)	4 (16.7)	0 (0)	0 (0)	17 (70.8)	0 (0)	0 (0)	18 (75.0)
**Median (IQR)**	7 (6–7)	5 (5–5)	3 (3–3)	6 (6–7)	5 (5–5)	3 (2–3)	6 (6–6)	5 (4–5)	2 (2–2)	6 (6–6)	5 (4–5)	1 (1–2)	6 (6–6)	4 (4–5)	1 (1–1)

***Note.– IQR*** *– interquartile range*

## Discussion

This experimental study aimed to evaluate the benefits of spectral shaping versus conventional and low-kV imaging for radiation dose reduction in unenhanced abdominal CT. We were able to ascertain the superiority of tin-filtered scans at 100 kVp over all other voltage settings, with advantages being most pronounced at the ultralow-dose level of 0.5 mGy. Notably, while the radiation dose of all investigated protocols was far below the national diagnostic reference standard for abdominal CT, i.e. 15 mGy [23], the dedicated ultralow-dose protocols realized a thirtyfold decrease, yet still providing sufficient image quality with spectral shaping at 100 kVp.

Although the results of this study concur with other publications that advocate spectral shaping for abdominal low-dose CT imaging [18, 21, 24, 25], protocols without tin filtration are still commonly applied by many radiologists as standard of care [26, 27]. Our findings are generally in line with an experimental study by Karmazyn et al., who reported that merely decreasing the tube voltage is insufficient for adult sized phantoms, especially in ultralow-dose imaging [28]. While this investigation lacked the assessment of additional tin prefiltration, our study included two different voltages settings with spectral shaping. Results indicate that the use of a tin filter at 150 kVp bears no decisive advantage over conventional or low-kV imaging. Therefore, in synopsis with the current literature, we postulate that this setting should rather be considered for obese patients or in cases where artefacts are to be expected due to the presence of metal implants [29–31]. Similarly, our findings do not support the superiority of 100 kVp versus 120 kVp, which has been suggested by some authors [27, 32].

On the other side of the spectrum, it is known that lowering the tube voltage leads to a redistribution of the effective radiation exposure with a higher surface dose and increased peripheral absorption of x-ray beams [33]. In an earlier patient study comparing a 120 kVp with a 90 kVp protocol, Nakayama et al. suggested a cut-off value of 70 kg for the latter to prevent a significant drop in SNR [34]. In contrast to similar studies comprising either Sn 150 kVp or Sn 100 kVp settings, our investigation bears the advantage of comprehensive comparisons between all relevant voltage settings at different dose levels for various tissues [24, 35]. Taking into account that none of the cadaveric samples was of obese stature, the reported advantages of Sn 100 kVp protocols regarding SNR and subjective image quality at all dose levels are in line with current literature [25, 27, 36]. As the detection of urinary calculi represents a high-contrast imaging task, suchlike examinations are ideally combined with dedicated low-dose and ultralow-dose scan protocols. In this context, prior studies have suggested dose saving potentials ranging from 30% to as much as 50% with regards to kidney stone imaging and by as much as 90% in chest imaging when combined with automatic tube current modulation and iterative reconstruction algorithms [25, 36, 37].

Some limitations must be considered for the present study: First, the body donors examined were of normal constitution, neither being morbidly obese nor cachectic. Since the selection of cadavers in this study was performed at random, the presence of urinary calculi could not be controlled. Despite this limitation, our study provides valuable insights into potential radiation dose reduction strategies in unenhanced abdominal CT, offering a basis for further investigations with larger patient samples. Second, the minimal applicable tube current with the employed dual-source scanner in a 120 kVp setting is 5 mAs. Although this setting was not selected in the current study, the limited tube current lowering potential could lead to a dose disadvantage in slim patients, while rendering a dose-comparable study at 150 kVp technically impossible. In clinical practice, however, it can be assumed that the automatic tube voltage selection prevents this constellation. Third, the dual-source scanner hardware and tin prefiltration technique are exclusive to one particular vendor. However, other manufacturers also rely on pre-patient beam filtration for hardening of the x-ray spectrum (albeit with different materials), enhancing the relevance and generalizability of our findings to a broader context. Finally, the present study was limited to investigations on energy-integrating detector technology. Novel photon-counting detectors may provide additional advantages when combined with dedicated low-dose protocols for abdominal imaging [35].

## Conclusion

Ultralow-dose scans of the abdomen, e.g. for the detection of urinary calculi, benefit from the application of spectral shaping with tin filtration at 100 kVp. Compared with low-kV or conventional imaging, both subjective and quantitative image quality were superior with Sn 100 kVp. In contrast, employing tin filtration at 150 kVp did not provide a similar advantage in the investigated sample of cadaveric specimens with normal body constitution.

## Data Availability

The datasets generated and/or analyzed during this study are not publicly available as CT data and DICOM headers contain patient information. Data can be obtained on reasonable request from the corresponding author.

## Abbreviations

CTDI_vol_: Volume computed tomography dose index
Sn: Marks a voltage setting with additional tin prefiltration
SNR: Signal-to-noise ratio
